# Development of Open-source Software and Gaze Data Repositories for Performance Evaluation of Eye Tracking Systems

**DOI:** 10.3390/vision3040055

**Published:** 2019-10-22

**Authors:** Anuradha Kar, Peter Corcoran

**Affiliations:** Department of Electrical & Electronic Engineering, National University of Ireland, H91 TK33 Galway, Ireland; peter.corcoran@nuigalway.ie

**Keywords:** eye gaze, eye trackers, fixations, data quality, performance evaluation, code repository, gaze dataset

## Abstract

In this paper, a range of open-source tools, datasets, and software that have been developed for quantitative and in-depth evaluation of eye gaze data quality are presented. Eye tracking systems in contemporary vision research and applications face major challenges due to variable operating conditions such as user distance, head pose, and movements of the eye tracker platform. However, there is a lack of open-source tools and datasets that could be used for quantitatively evaluating an eye tracker’s data quality, comparing performance of multiple trackers, or studying the impact of various operating conditions on a tracker’s accuracy. To address these issues, an open-source code repository named GazeVisual-Lib is developed that contains a number of algorithms, visualizations, and software tools for detailed and quantitative analysis of an eye tracker’s performance and data quality. In addition, a new labelled eye gaze dataset that is collected from multiple user platforms and operating conditions is presented in an open data repository for benchmark comparison of gaze data from different eye tracking systems. The paper presents the concept, development, and organization of these two repositories that are envisioned to improve the performance analysis and reliability of eye tracking systems.

## 1. Introduction

Gaze data quality refers to the validity of the gaze data measured and reported by an eye tracker [1]. The most common method of representing gaze data quality is by specifying gaze estimation accuracy, which refers to the difference between the true and the measured gaze positions [2]. There currently exists significant diversity in gaze accuracy measures as described in reference [3], which leads to ambiguity in interpretation of the quality of gaze data from different eye tracking systems and difficulty in comparison of two or more eye trackers. Moreover, with the growing applications of gaze information in consumer devices like augmented and virtual reality, smartphones, and smart TVs [4,5,6,7] the eye trackers used in such applications need to be thoroughly evaluated to ensure the high quality and consistency of their gaze data outputs. This calls for the development and adoption of homogeneous metrics for reporting gaze accuracy and a consistent set of methods for complete characterization of eye trackers’ data under different operating conditions [8]. There are several software tools [9,10,11,12,13] that have been developed over the years by gaze researchers as well as eye tracker manufacturers for gaze data analysis. The general focus of these software is toward determining eye movement characteristics (i.e., fixations, scanpath, saccades) and studying eye movement relationships with human cognitive process, such as creation of attention maps, understanding regions of user interests, and visual search patterns. Also, a range of gaze datasets have been developed so far by gaze researchers, which are either aimed at building of new gaze estimation algorithms or toward cognitive studies, visual saliency research, and scanpath analysis. However, gaze datasets that contain gaze and ground truth data collected under different operating conditions of an eye tracker, from multiple user platforms, are not yet publicly available.

In this paper, two open-source code and data repositories are presented that are targeted specifically toward in-depth analysis and comparison of eye gaze data quality from generic eye trackers. These repositories are (1) the GazeVisual-Lib repository of software resources hosted on GitHub and (2) the NUIG_EyeGaze01 gaze data repository hosted on Mendeley data. This paper describes the creation, organization and usage of these two repositories that are aimed towards standardized evaluation of the performance of generic eye-trackers. These repositories can benefit gaze researchers, developers of gaze-based systems and applications, and generic users by providing them easy-to-use methods for quantitatively evaluating gaze data outputs from an eye tracker, compare quality of two or more trackers or user platforms. The key features of these two repositories are summarized in Section 1.1 and Section 1.2 below.

The motivation behind developing the GazeVisual-Lib software repository is that it could be used by gaze researchers to analyze gaze data and answer critical questions related to gaze data quality. For example, what are the performance limits and tolerances of a given eye tracker? How much is an eye tracker’s accuracy affected when operating under non ideal operating conditions? Which operating conditions affect the tracker’s performance in a particular use-case? How can two gaze datasets, or the performance of two eye tracking systems, be compared quantitatively? What are the performance bottlenecks of individual algorithms? How can gaze error patterns be detected and predicted? The software resources provided in the GazeVisual-Lib repository can help any generic user or an eye gaze researcher to find answer to these questions with minimal programming effort. 

The motivation for developing the NUIG_EyeGaze01 data repository is to present gaze datasets collected under unique and challenging operating conditions which are not usually available to gaze researchers. The gaze data within the repository has been collected from a high-resolution eye tracker under carefully designed operating conditions so that best- and worst-case performance characteristics of an eye tracker under the influence of these conditions may be studied. These gaze datasets can help researchers to compare the variation in the data quality of multiple eye trackers, determine anomalous gaze data types, and study a tracker’s reliability and system characteristics under unconstrained operating conditions. 

### 1.1. GazeVisual-Lib: An Open Software Repository for Eye Tracker Data Evaluation

This paper describes the GitHub code repository named GazeVisual-Lib that contains the source codes for a complete GUI application tool and a range of numerical and visualization methods for quantitative and visual exploration of eye gaze data quality. A major component of the GazeVisual-Lib repository is the source code of a desktop GUI software application named GazeVisual, which takes in raw eye gaze data and implements several accuracy metrics and a range of visualizations to study gaze data quality. It also has methods to interface the GUI with an eye tracker for live gaze data collection [14]. Multiple videos are provided in the repository that show how to use the software to upload gaze data and derive results and visualizations. Apart from this, in the repository, there are codes in different sub-directories that could be used for (a) estimating gaze accuracy in angular resolutions as the difference between input gaze and ground truth data coordinates, (b) metrics and visualizations for exploration of gaze data quality [8], (c) de-noising and outlier removal from gaze data, and (d) augmentation of a gaze (fixation/scanpath) dataset by seven different methods. The GazeVisual-Lib repository is hosted on GitHub and accompanies full documentation and guidance on the use of individual repository components. The GitHub repository can be found at github.com/anuradhakar49/GazeVisual-Lib.

This paper provides details on how to use the GazeVisual-Lib repository, installation of the dependencies i.e., Python libraries (www.python.org) required for running the codes from the repository, and practical illustrative examples that would guide a user to run the GazeVisual GUI tool. Also, all the Python codes are made available as Jupyter notebooks within the GitHub repository, so that any user can run these and adapt these resources easily. 

### 1.2. NUIG_EyeGaze01: An Open Gaze Data Repository

In addition to the open coding resources, a new eye gaze dataset is presented in this paper, named NUIG_EyeGaze01 (Labelled eye gaze dataset). This dataset is created through dedicated experiments, using data from a high resolution eye tracker while it operated on three different eye tracking platforms—a desktop, a laptop, and a tablet—under a wide range of operating conditions such as variable user head poses, user distances, screen resolutions, and platform poses. The gaze data files are made available publicly and could be useful to gaze researchers for benchmark comparison of performance of other eye trackers, for building advanced gaze data evaluation metrics, and also for understanding gaze error patterns caused by the different operating conditions mentioned above. The NUIG_EyeGaze01 dataset is hosted on Mendeley Data, which is an open data repository and may be found in the following link: https://data.mendeley.com/datasets/cfm4d9y7bh/1.

In this paper, details on the data collection process for creation of the NUIG_EyeGaze01 dataset and the dataset organization is provided. The contents of the collected gaze data files are discussed along with sample data presentation from the various experiments done for the data collections. Also, a sample Python code snippet is provided in this paper that may be used to read from the CSV data files in the open dataset, so that researchers can readily use these datasets and extract and manipulate the information in them. Finally, the utility and significance of the dataset and the coding resources toward gaze research are discussed. 

### 1.3. Scope and Organization of the Paper

The scope of this paper is focused around discussing the organization and contents of the two code and data repositories described in Section 1.1 and Section 1.2. This paper describes the components of the GazeVisual-Lib repository along with detailed instructions on how these resources maybe used with minimum programing effort. This is done so that readers can understand the purpose, contents, and implementation of the GazeVisual-Lib repository, and it can be readily useful to the interdisciplinary gaze research community for evaluation of gaze data quality. It may be noted that the mathematical derivation of the metrics, visualizations, and concept of the GazeVisual GUI application (present in the GitHub repository) have been discussed in details in our previous papers [8] and [14], which provide the scientific background for the coding resources presented in the repository. In a similar way, this paper describes the content and structure of the NUIG_EyeGaze01 data repository with details on each data file, their columns and file naming conventions and sample usage. These would ensure that the collected datasets may be easily used by vision researchers. The philosophy behind the gaze data collection process has been discussed in [8].

The paper is organized as follows: Section 2 presents a literature review on contemporary gaze data evaluation software and publicly available gaze datasets. Section 3 describes the structure and contents of the GazeVisual-Lib repository, and Section 4 presents the details of the NUIG_EyeGaze01 data repository. Sub-Section 4.4 presents discussions and analysis of the collected datasets. Appendix A presents the installation instructions for the various libraries required to run the GazeVisual-Lib coding resources, and Appendix B contains a series of gaze data plots created using data from the NUIG_EyeGaze01 repository.

## 2. Previous Works on Open-Source Gaze Data Analysis Software and Gaze Datasets 

Eye-tracking has found applications in fields such as neuro and cognitive sciences, psychology, human-computer interactions, consumer electronics, and assistive technologies [15]. Performance of eye trackers are judged based on their accuracy (in degrees of angular resolution) and it is affected by physiological limitations, tracker setup geometry, or due to type of calibration techniques used in them [16]. Works such as references [2] discuss the evaluation and comparison of several commercial eye trackers. In reference [5], an open-source Matlab toolkit is presented that can be interfaced with a commercial (Tobii EyeX) tracker. The paper evaluates and reports the eye tracker’s performance in terms of angular accuracy, precision, latency, and sampling frequency. In reference [6], the performances of three wearable trackers, from Pupil Labs (120 Hz), SMI, and the TobiiPro, are compared in terms of their accuracy under multiple viewing conditions. In reference [2], the accuracy and precision the Eye Tribe tracker is compared with the SMI tracker. The work concluded that the selection of software to record and process the data are significant in obtaining high accuracy results from an eye tracker. 

There are several open-source software packages and toolboxes that have been developed for recording and analyzing gaze data, for example ETCAL [7], Pygaze [10], GazeParser [17], EyeMMV [18], and GazeAlyse [19] to name a few. PyGaze is an open-source software package in Python language which is built for creating eye tracking experiments, e.g., for presentation of visual stimulus and collection of user response via keyboard or mouse. It also allows online detection of eye movements and supports a wide range of commercial eye trackers. Another Python based open-source library is GazeParser which was developed for low-cost eye tracking, gaze data recording and analysis. It captures images from a camera to record eye position and subsequently performs calibration, synchronization of stimulus presentation along with recording and analysis of eye movements. Eye Movements Metrics & Visualizations (EyeMMV) is a toolbox built using MATLAB for eye movement analysis. It contains functions for identifying fixations, heatmap, and scanpath visualizations and region of interest analysis. Another Matlab-based toolbox is GazeAlyze, which does analysis of eye movement data, e.g., detecting and filtering artefacts, generating regions of interest, and visualizations such as path plots and fixation heat maps. There are also functions for correcting eye movement data due to the head position changes. The EMA toolbox [20] is implemented in Matlab for eye movement analysis and can parse gaze data from eye trackers like SR Research, SMI (RED 250), and Tobii EyeX. This toolbox allows for data conversion from normalized to pixel to degrees, determination of saccades and their kinematics, and creating saliency maps. Another toolkit named Pytrack [21] is built for analyzing and visualizing eye tracking data, feature extraction with respect to blinks, fixations, saccades, micro-saccades and pupil diameter, generate gaze heat map, micro-saccade position, and velocity plots.

ETCAL [7] is a recent development among open-source gaze research tools, and it is a library that provides a common platform to implement a range of calibration procedures to determine gaze points from raw eye movement recordings. The library contains algorithms for preparation and optimization of calibration models and automatic detection of gaze targets for implicit calibration scenarios. ETCAL is a useful tool for researchers who work with different calibration scenarios or want to build their own eye trackers, compare different calibration algorithms and data quality.

It may be observed that most of software developed so far for eye trackers aim towards exploration of eye movement characteristics (detecting fixations, scanpath, saccades, eye movement speed, direction, duration), studying eye movement and their relationships with human behavior (such as building attention maps), deriving regions and sequence of interests, and analyzing cognitive processes. However, only a few software tools (for example, ETCAL [7]) exist that are designed for quantitative evaluation and visualization of gaze error characteristics, e.g., for estimation of gaze error statistics and distributions and comparison of gaze errors collected under different operating conditions (or error sources). Therefore, in this paper, a new open-source repository of Python-based software tools is presented that can be used for the in-depth analysis of the gaze error characteristics that is collected from any eye tracker, irrespective of the tracking platform, hardware, or algorithm.

With respect to eye gaze datasets, there currently exists a multitude of them, and more are being developed by researchers to cater to individual research problems. A survey of gaze datasets was made, and it was observed that existing gaze datasets can be broadly classified into two types: the ones used for building and testing gaze estimation algorithms, and the others that are used for modelling and validating user attention patterns and cognitive processes. Table 1 shows the results of this survey and presents the details of several datasets that have been developed for building and testing gaze estimation algorithms. Table 2 presents the datasets developed for saliency and cognitive studies. These datasets have been developed with users looking at a series of images while their eye movements/images/videos are recorded. The collected eye movement data is then used for building and validating cognitive studies, visual attention patterns, saliency models, etc.

It is observed that typically gaze datasets include eye images/video, eye corners, iris, blink rate, eye closure, fixation or smooth pursuit data. Some include head pose information, while datasets are captured under “free-head motion,” i.e., the exact angular positions of the user head are not known. Some datasets include conditions such as users with/without glasses, change in illumination and background, varying race, age, etc. In this work, a new eye tracking dataset is built comprising of gaze data from three different user platforms, specifically for benchmark evaluation of eye trackers operating under unconstrained operational scenarios and is described in Section 4 of this paper.

## 3. Description of the GazeVisual-Lib Code Repository

### 3.1. Organization of the Repository

The GazeVisual-Lib repository is hosted on GitHub and contains numerical and visual methods implemented as Python codes that can be used to evaluate and compare data quality and characteristics of generic eye trackers. The methods require gaze data samples from an eye tracker, ground truth locations, and values of setup variables like user-tracker distance, size, and resolution of the display screen where gaze was tracked. Table 3 presents an overview of the repository.

### 3.2. Functionalities of the GazeVisual-Lib Repository Components

**Figure 1:**
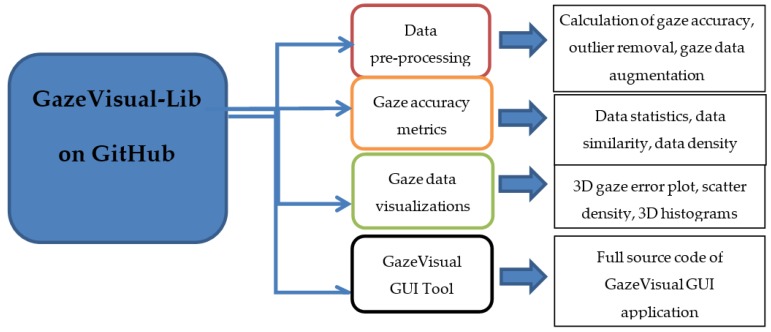
Organization of the GazeVisual-Lib code repository on GitHub.

**Figure 2:**
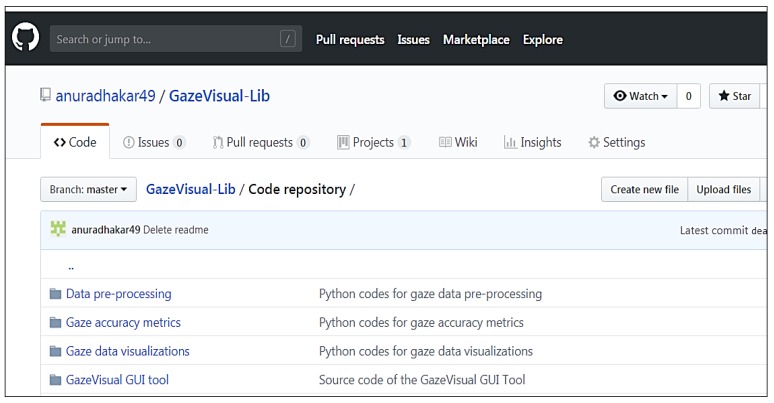
View of the GazeVisual-Lib code repository on GitHub.

The GazeVisual-Lib repository provides easy-to-use gaze data evaluation resources for free use, modification, and upgradation by eye gaze researchers and engineers. As shown in Figure 1, the contents of the repository are organized into multiple sub-directories, each containing a set of codes written in the Python language. The Python codes and supporting information for using the numerical methods and visualizations are included in the repository in different folders (Figure 2). The details about the contents of these folders and their functionalities are provided below. For running these codes, a user must have Python 2.7 along with libraries like Python libraries like Numpy, Matplotlib, Tkinter, Pygame, Statsmodels, and Seaborn installed.

#### 3.2.1. “Gaze Data Pre-Processing” Folder

**Figure 3:**
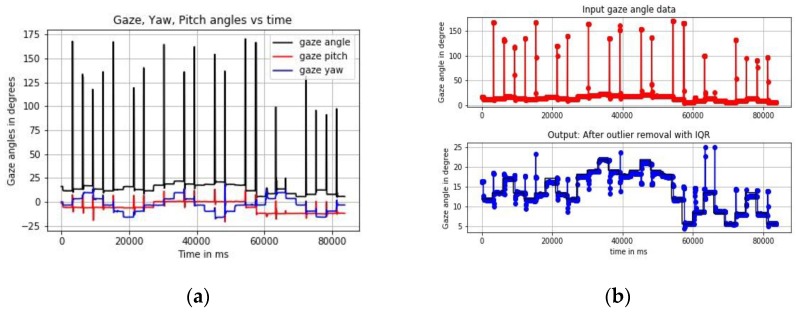
Outputs from using GazeVisual-Lib codes on eye gaze data (**a**) gaze angle, yaw, pitch values as a function of time (**b**) outliers removed from gaze data using IQR method.

In this folder, there are three Python or .py files, also combined in a Jupyter Notebook or .ipynb file (named: Data pre-processing.ipynb), which are meant to perform the following functions:

(1) Raw data conversion and calculation of accuracy: The main_proc.py file in this folder estimates gaze angular and gaze yaw and pitch accuracies from raw gaze data samples and ground truth data. The output is a CSV file (user_data_proc.csv), which contains several gaze angular variables (gaze yaw, pitch, frontal angle) and gaze accuracy values which are the angular differences between estimated gaze locations and stimuli locations [8].
(2) Outlier removal: Gaze data is almost always corrupted with outliers and it is impossible to observe any error patterns until outliers are removed. The outlier_removal.py file implements three different outlier detection and removal strategies which are 1D Median filtering, median absolute deviation and interquartile range method [8].
(3) Data augmentation: Six different methods for synthesizing gaze angular data variables from an original gaze data file are presented in the code named data_augmentation.py. The methods include addition of white and colored noise, data interpolation, time-shifting, data convolution with cosine kernels, and a combination of these. Augmented gaze datasets may be used for purposes like the use of machine learning algorithms [48] to model gaze data patterns of a tracker, modelling of scanpath and search patterns. Sample output plots from the main_proc.py and the outlier_removal.py codes are in Figure 3a,b.

#### 3.2.2. “Gaze Accuracy Metrics” Folder

In this folder, there is a sample gaze data file (user_data_proc.csv, which is produced from the main_proc.py program) and three files (data_statistics.py, data_similarity.py, spatial_density.py) that may be used to compute gaze data statistics, similarity between gaze datasets, and gaze error spatial density on the display screen where gaze was tracked. The data_statistics.py file calculates mean gaze angular error (over the number of gaze data points), standard and median absolute deviation, confidence intervals, and Z-score of the gaze angular error from an input gaze dataset. The file data_similarity.py computes the similarity between gaze data, e.g., from different eye trackers or experiments. The similarity calculation is based on correlation, intersection, and Bhattacharya distance [8] computed on histograms of two gaze datasets. The scatter_density.py file helps to create a gaze data density plot in which raw gaze data is plotted as data point clusters and color-mapped according to point densities, which helps to study gaze data patterns and detect anomaly. All these are combined in the Jupyter Notebook named “Gaze accuracy metrics.ipynb” kept within the folder.

#### 3.2.3. “Gaze Data Visualizations” Folder

In this folder there is a sample gaze data file (user_data_proc.csv, output of the main_proc.py) and 4 python codes (combined in the Gaze data visualizations.ipynb file) that implement various visualizations [8]. The file 3D_plot.py creates a 2D grid of on-screen locations and produces a 3D plot of the magnitude of gaze errors (along *Z*-axis) as a function of X and Y dimensions of the display screen. These plots help to diagnose gaze error levels over the display area.

**Figure 4:**
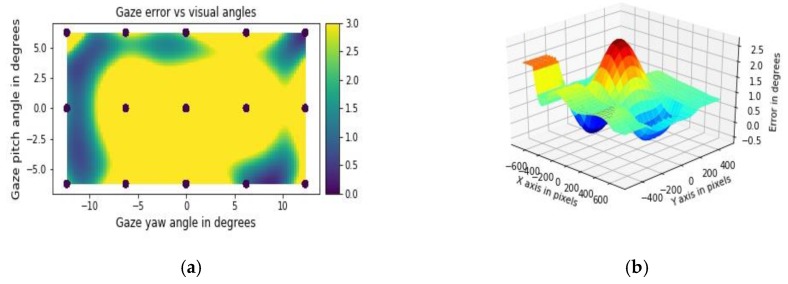
Outputs from using GazeVisual-Lib codes: (**a**) 2D surface distribution of gaze errors as a function of visual angles; (**b**) 3D plot of gaze errors as a function of display dimensions in pixels.

The eccentricity.py file plots create a 2D surface plot of gaze error levels mapped with respect to visual angle values on the display screen, using data from the DIFF GZ” and “YAW DATA” and “PITCH DATA” columns, respectively, from the user_data_proc.csv file. This plot program may be used to study how gaze errors vary with visual angles, especially if user distance from the tracker is increased or decreased. For shorter distances, gaze errors are usually more sensitive to visual eccentricities, whereas gaze errors for long distances (e.g., at 80 cm) show less sensitivity to eccentricities [8]. The file 3D_histogram.py plots stacked 3D data distributions using data from two or more trackers or experiments. It helps to understand and compare data patterns and gain insight into data characteristics, e.g., where error values are concentrated or presence of data extremes. Sample output plots from the eccentricity.py and 3D_plot.py are shown in Figure 4a,b.

#### 3.2.4. “GazeVisual GUI Tool” Folder

**Figure 5:**
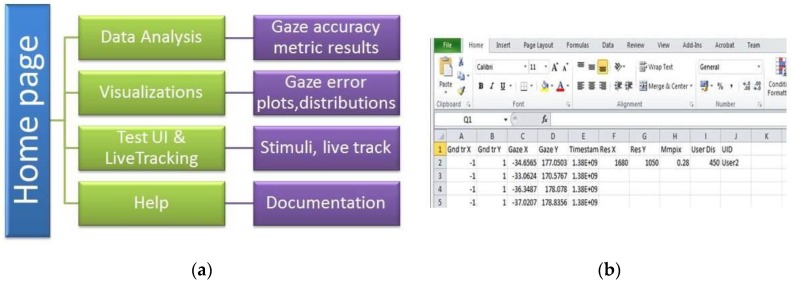
(**a**) Architecture of the GazeVisual software; (**b**) Input data format (CSV) for the software.

**Figure 6:**
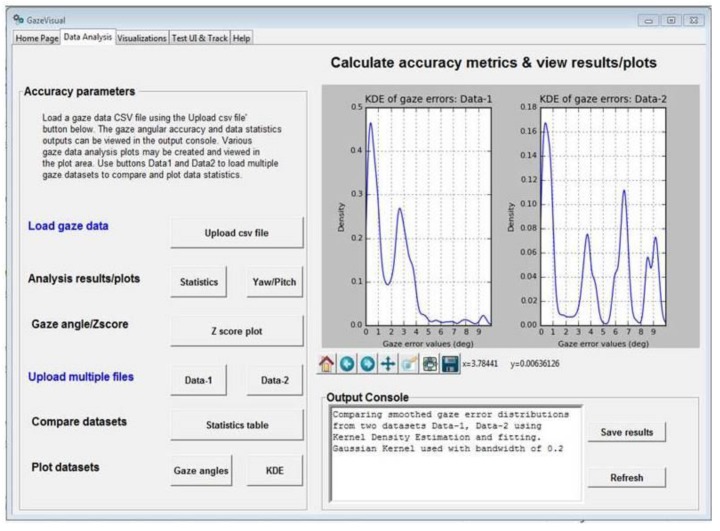
View of the “Data Analysis” window of the GazeVisual software.

**Figure 7:**
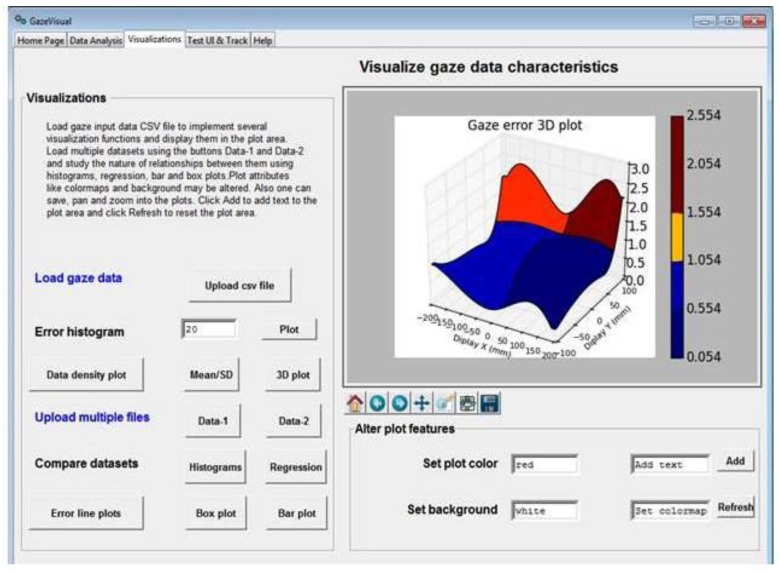
View of the “Visualizations” window of the GazeVisual software.

This folder contains the source code (contained in the files gazevisual_v101.py, gazevisual_v101.ipynb) for the GazeVisual GUI application software. This software is designed for quick, easy, and in-depth evaluation of eye tracker data through a suite of statistical and visualization functions incorporated in it. GazeVisual comes in the form of a graphical user interface (GUI) and contains a range of functions to input and process gaze data files and produce various gaze accuracy metric results and visualizations. It can generate visual stimuli and can also be directly interfaced with an eye tracker to collect gaze data samples. It is entirely built in Python language using several data analysis and graphics libraries. The architecture of GazeVisual software is shown in Figure 5a and views of the software are in Figure 6 and Figure 7.

The GazeVisual software is comprised of four independent windows containing a range of functions incorporated in it that are aimed toward gaze data evaluation. Input data format for the GazeVisual software is shown in Figure 5b. It is comprised of columns for raw gaze and ground truth data coordinates and input variables like display screen resolution and pixel pitch of the display (µ) [8] and user distance from the tracker. Two sample gaze data files that can be input to the GazeVisual software are provided in the GitHub folder. The GazeVisual software can be compiled as a generic Python program/IPython Notebook to produce the GUI application. Following this, the input gaze data files can be uploaded to the software using the “Upload csv file” button, and then the rest of the software functionalities may be implemented using the other GUI buttons. Outputs of the software include gaze accuracy values, error statistics, plots of error numerical and spatial distributions, and comparison of two gaze datasets [14].

The “Data Analysis” window provides functions for estimation of gaze angular variables, accuracy (in degrees), gaze error statistics, and distributions using a single gaze data file and allows comparison of these parameters for two gaze data files. The “Visualizations” window can be used to plot gaze error histograms and 3D spatial distributions from a gaze data file. It can also take in two gaze data CSV files and compare their characteristics by creating correlation/regression/box plots. The Test UI and LiveTracking window can create static and dynamic stimuli for data collection from an eye tracker and also interface GazeVisual with an eye tracker for direct data collection.

The GazeVisual GUI application has been tested with data from two remote eye trackers and a head mounted eye tracker and is seen to produce consistent results [14]. Its input requirements are (a) input gaze data (x, y coordinates) and corresponding ground truth data coordinates, (b) input gaze and ground truth data coordinates should have their origin at display center, (c) input data is arranged in the format shown in Figure 5b, (d) the data is free from non-numeric or NAN values, and (e) gaze and ground truth data must have same lengths (number of data rows). Sample input data files for testing this software may be found in the GitHub folder (named “usr1_45_gazedata.csv”). GazeVisual can be compiled and run as a desktop application on any operating system having Python 2.7 with libraries such as Tkinter, Pygame, Statsmodels, and Seaborn installed.

The GazeVisual GUI tool could be extended by gaze researchers in various ways. For example, in Figure 7, a single dataset is used to plot the figure, and the utility of this is to study gaze data characteristics from single person or experiments. However, functions for plotting the mean values of gaze errors and error patterns for different persons could be a valuable feature that could be added to the software in future. Although this feature is not present in the current version of GazeVisual, it is always possible to extend the software features to include more functions such as for uploading multiple datasets, numerical functions e.g., for data filtering and outlier removal. Also, common APIs could be developed to interface the GUI will multiple eye trackers. This is the utility of making the software and codes open resources that are freely available for modification.

#### 3.2.5. Steps for Using the Gaze Data Evaluation Methods in GazeVisual-Lib for Analysing Gaze Data

The GazeVisual-lib is hosted on GitHub and can be found in the following web-address: github.com/anuradhakar49/GazeVisual-Lib. It is released under the GNU-GPL v3.0 license, which allows the users to run, share, and modify the software and the source codes in the repository.

**Figure 8:**
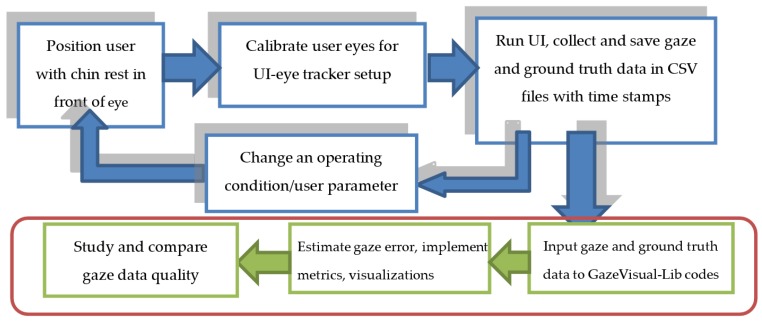
Workflow to implement gaze data evaluation methods of the GazeVisual-Lib repository.

An experimental setting for using this code repository and its components is schematically shown in Figure 8. For using the gaze data evaluation methods, first of all, a sample of gaze data from one or more participants is required [8]. To collect gaze data, (a) a user has to sit in front of the eye tracker under test, which is mounted on a computer screen, and the user eyes are calibrated [49]. (b) The user is presented with visual stimuli, and the eye tracker under test should record the gaze coordinates of the user as the user gazes at the stimuli points. (c) The gaze data from the eye tracker is to be saved in CSV format. The ground truth data comprising the screen coordinates of the visual stimuli points appearing during gaze data collection are also to be saved in a separate CSV file.

The raw gaze and ground truth data collected in the above manner can then be used as inputs to the codes in the repository and the GazeVisual software application. Details on how the gaze and ground truth data files may be used with the repository codes to estimate gaze accuracy, and successively implementing other gaze data evaluation methods is provided within the repository.

To run the codes in the GazeVisual-lib code repository, users must have Python 2.7 and the different Python libraries installed (installation commands may be found in Appendix A). All the codes are in the “Code repository” folder (Figure 2 above), which has several “README” files in various sub-folders that provide details about how to format an input data file and use it with the codes. The README file of the root folder is the main documentation for the repository, which has details about how the codes may be run and the current repository version. Researchers should check this main README file to learn about current and subsequent version updates. Also examples showing the workings of the GazeVisual GUI software may be found in the videos S1 and S2, mentioned in the “Supplementary Materials” section of this paper below. Links to the videos are https://www.youtube.com/watch?v=mPGlw711BCA (Video 1, showing Visualization functions) https://www.youtube.com/watch?v=sir_qZmvGME (Video 2, showing Data Analysis functions).

Gaze data from any source (e.g., eye tracking device, application, or algorithm) must be formatted as per the instructions in the README files and used first with the main_proc.py in the “data pre-processing” sub-folder to produce an output CSV file. The rest of the code functions are based on this output CSV file. Similarly, to use the GazeVisual GUI tool, users should copy and save the GazeVisual_v101.py file into any directory of their computer, run it as regular python codes, and make sure all the imported libraries are pre-installed. Sample CSV files to test the codes and understand the data format are in each sub-folder of the repository. Links to sample videos showing the operation of the GazeVisual GUI tool are in the “sample videos” file of the GUI folder.

The gaze data evaluation methods in GazeVisual-Lib are based on calculations using data from both eyes and centralized gaze coordinates. If any eye tracker provides only monocular data or only centralized gaze coordinates without left/right eye position values, then the repository codes can still be used for gaze angle and accuracy calculations after minor changes. The metrics and visualizations in the repository will still work, but the results may vary from the case when binocular data is used.

#### 3.2.6. Installing the GazeVisual-Lib Components

For using the GazeVisual-Lib code repository, the repository has to be downloaded from GitHub manually or cloned using git. For cloning a Git bash shell (on Window) or terminal (OSX, Linux) and the repository is to be cloned by the command git clone https://github.com/anuradhakar49/GazeVisual-Lib.git.

Prior to using the repository components, Python 2.7 must be installed on the computer, which can be easily done through the Anaconda distribution for Windows, MAC, and Linux operating systems (https://www.anaconda.com/distribution/). After installing Python 2.7 through the Anaconda installer, the Anaconda prompt—which is a terminal application for installing Python libraries—is to be opened, and the libraries required for running the GazeVisual codes are to be installed. Table A1 in Appendix A lists the names of the special libraries used in the repository codes (libraries like math, csv, numpy, os, sys, etc. are pre-installed in Python) and the commands that have to be entered to the Anaconda Prompt to install them in their latest versions. For persons using pip for Python to install the libraries, the corresponding commands are also provided.

In addition to the individual Python scripts, all the methods within the repository have been made into Jupyter notebooks, in which the users do not need to install the libraries separately. A Jupyter notebook is a web-based interactive environment for the development of Python codes, which can be used for data analysis and visualization over a browser. This makes it very simple for users to run, view the outputs, and share the GitHub repository codes. All the codes in the GazeViusal-Lib repository can thus be run as Python scripts or as IPython notebooks. Jupyter can be opened by typing on Anaconda prompt (or terminal) the following: “jupyter notebook”.

#### 3.2.7. Running the GazeVisual-Lib Codes and the GazeVisual GUI Application

**Figure 9:**
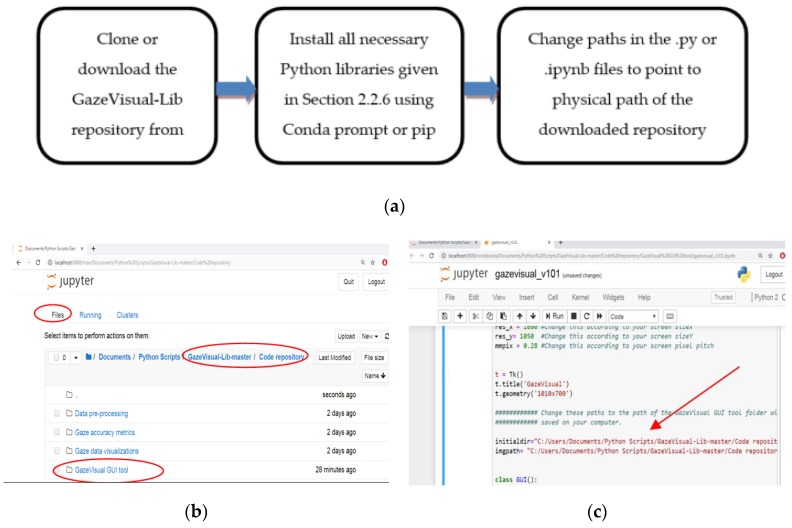

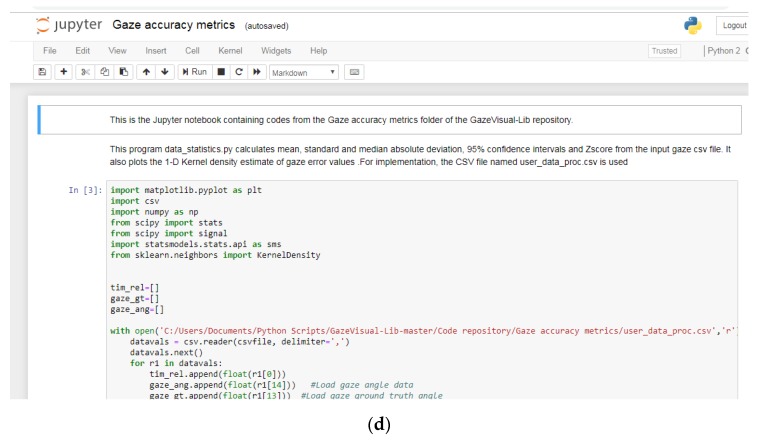
(**a**) Flowchart for running the GazeVisual-Lib codes. (**b**) Opening the Jupyter Dashboard to locate the downloaded GazeVisual-Lib folder. (**c**) Running the GazeVisual GUI code on Jupyter (arrow shows the paths which have to be changed). (**d**) View of the Jupyter notebook for the Gaze accuracy metrics folder.

Running the codes of the GazeVisual-Lib repository is a three-step process (Figure 9a). First, the repository has to be cloned or downloaded to a user’s computer. After this, the necessary libraries need to be installed as described above. Finally, the Python (.py) files or Jupyter notebooks (.ipynb files) are to be opened, and within, them the path variables (also mentioned in comments within the codes) have to be changed so that they refer to the actual physical location of the downloaded repository folder and its sub-folders on the user’s computer. After this, the repository codes can be run as normal Python script files or Jupyter notebooks. The CSV data files in the downloaded repository need to be checked to ensure that they contain column-wise data as shown on page, or they need to be formatted into columns using the “text to columns” function of MS Excel.

An illustrative example of running the Jupyter notebook for the GazeVisual GUI application tool is described here. To open up the Jupyter application, the steps described in the last section should be followed and thereafter the Jupyter Notebook Dashboard will open up in the web browser address: http://localhost:8888 as shown in Figure 9b. On the Dashboard, the Jupyter Notebook App can access the files within its start-up folder, so a user has to navigate and find the folder where the .ipynb files are stored. As in Figure 9b, the main repository folder downloaded from GitHub named GazeVisual-Lib-master is seen under “Files” tab, which is where all the .ipynb files can be accessed.

The user has to click on “GazeVisual-Lib-master,” then click on the folder “Code repository” within it, and then click the folder name “GazeVisual GUI tool” to reach the GazeVisual_GUI.ipynb file. Then, to run this file, the user has to click on this file, upon which the notebook containing the code for the GUI application will open up in a new browser tab, as shown in Figure 9b. Next, the path addresses “initialdir” and “imgpath” have to be changed so that they point to the physical location of the “GazeVisual-Lib-master” folder on the user computer. Finally, to run the GUI code, the whole notebook has to be run by clicking on the menu Cell → Run All (Figure 9c). To restart the kernel and run the code afresh, the user has to click on the menu Kernel and then click Restart.

## 4. Description of the NUIG_EyeGaze01 Gaze Data Repository

There are currently no publicly available eye gaze datasets that allow benchmark comparison and analysis of gaze data quality from two or more eye trackers. Also, there are no datasets that can be used to study gaze error patterns caused by various external operating conditions (or error sources) like head poses, user distances, or platform poses. While there exist plenty of eye gaze datasets containing eye images and videos or fixations and scanpath, none of them contain fixations and corresponding ground truth data collected from more than one eye tracking platform on different display resolutions. Without high-quality gaze data collected under measured variations of different such operating conditions (or error sources), no objective or practical comparisons of performance of new and/or existing gaze tracking systems can be made.

Considering these factors, a rich and diverse gaze dataset, using the eye tracking data collected through dedicated eye tracking experiments conducted under wide range of operating conditions, is therefore built and presented in an open data repository. The dataset is named NUIG_EyeGaze01 (Labelled eye gaze dataset) and is hosted in the Mendeley open data repository with the doi:10.17632/cfm4d9y7bh.1. The link to the dataset is provided in Section 1.2. This is a new kind of gaze data set, collected from three user platforms (desktop, laptop, tablet) under the influence of one condition at a time. Using this dataset, the impact of different operating conditions may be observed and quantitatively compared. The conditions include fifteen different head poses, four user distances, nine different platform poses, and three display screen size and resolutions. Each gaze data file is labelled with the type of operating conditions under which it was collected.

### 4.1. Description of the Gaze Data Collection Process

**Figure 10:**
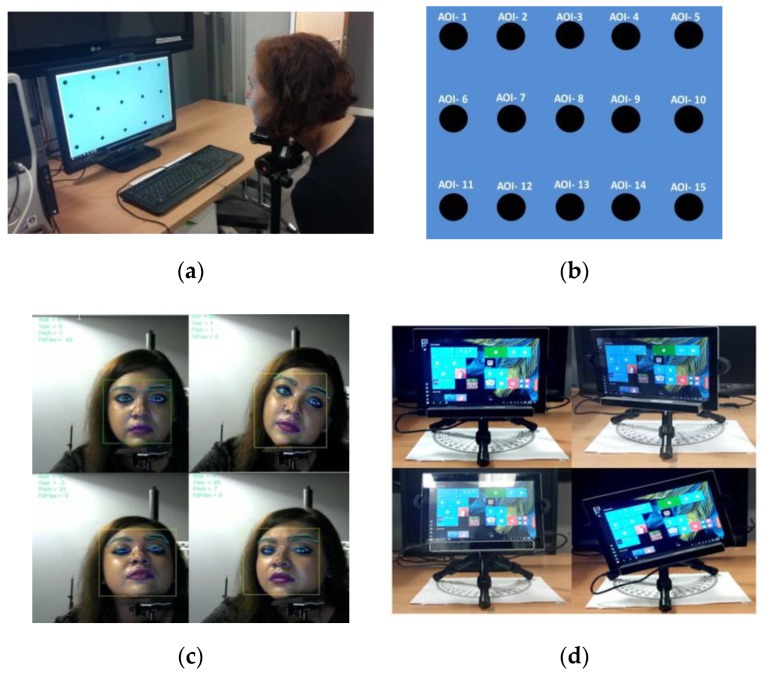
(**a**) Positioning of a participant for an experiment session (**b**) layout of the stimuli points for data collection. AOI stands for area of interest. Different (**c**) head poses and (**d**) tablet poses under which gaze data was collected.

The gaze data collection setup for creating the NUIG_EyeGaze01 dataset included a commercial eye tracker mounted on a desktop computer, a laptop and a tablet whose specifications are provided in Table 4. A Tobii EyeX eye tracker was used for gaze estimation, and an Eyetribe tracker was used for pilot data collection. Participants were seated in front of the tracker-screen setup, and their chin was fixed with a chin rest (Figure 10a). Prior to each data collection session, the eye tracker was calibrated with its own calibration software (six-point calibration). After calibration, a visual stimulus interface (Figure 10b) was presented to the participants [4], and they were asked to gaze at the specific stimuli targets that appeared on the display screen as their gaze was recorded by the eye tracker. For each experiment session, the following gaze data parameters were estimated for each user: (a) gaze positions data vs ground truth data (locations of stimuli) in pixels and millimeter (b) gaze yaw, pitch angles vs time, and corresponding ground truth yaw, pitch angles vs time (ms);(c) gaze primary angular error, yaw error, and pitch error for each stimuli position and time point.

For gaze data collection under variable operating conditions of the eye tracker, a series of gaze data collection experiments were done on the desktop, laptop and tablet platforms using the eye tracker. These experiments included (a) user distance experiments where users were seated at 50, 60, 70, or 80 cm from the tracker. This was done for the desktop, laptop and the tablet platforms (b) head pose experiments where a user had to position their head at certain fixed head pose angles while their gaze data was collected (Figure 10c). This was done only for the desktop platform (c) platform pose experiments, where the eye tracking platform or tablet was oriented at certain fixed tablet pose angles Figure 10d while user gaze data was collected. This was done only for the tablet platform. Further details about the participants, experimental setup and variables may be found in Table 4. Table 5 provides the details about the contents of each CSV data file contained within the repository and a description of the data columns.

**Figure 11:**
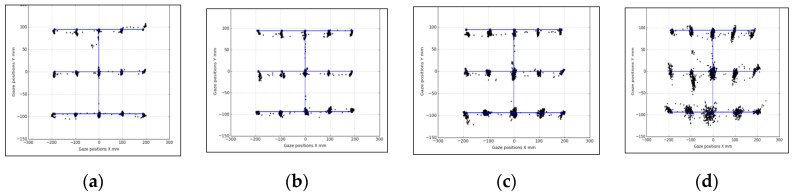
Data from user distance experiments: Desktop (**a**) 50 cm, (**b**) 60 cm, (**c**) 70 cm, and (**d**) 80 cm.

**Figure 12:**
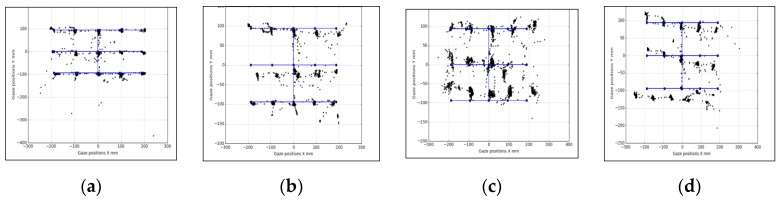
Data from head pose experiments: (**a**) neutral, (**b**) roll +30, (**c**) pitch +20, and (**d**) yaw +30 degrees.

**Figure 13:**
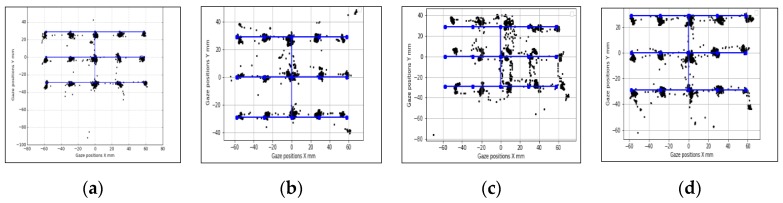
Tablet pose experiment data: (**a**) neutral, (**b**) roll +20, (**c**) pitch +20, and (**d**) yaw +20 degrees.

In Figure 11, Figure 12 and Figure 13, samples of eye gaze data overlapped on ground truth locations of the stimuli from each of these experiments are provided. Gaze data is in black and ground truth data are in blue. It is seen that data from different experiments look consistent but are affected by variable levels of outliers, which is why the outlier removal methods are provided in the GazeVisual-Lib repository. All these data, along with time stamps, were written in comma separated values (CSV) format for each user and each experiment session. Gaze data plots from multiple participants for the different operating conditions may be found in Appendix B of this paper.

### 4.2. Organization of the NUIG_EyeGaze01 Gaze Dataset on Mendeley Data

**Figure 14:**
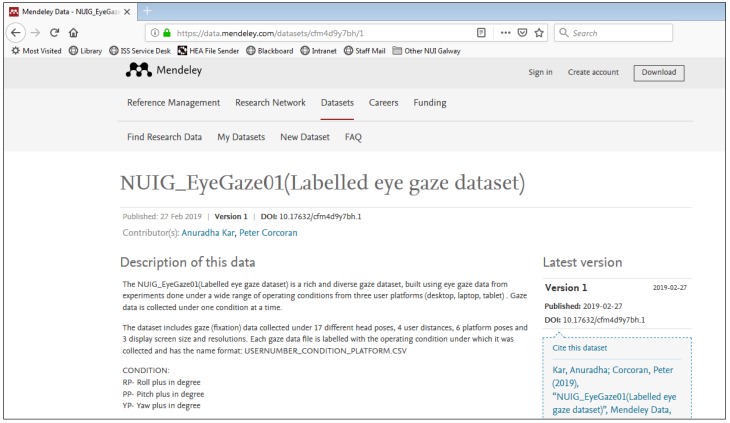
Snapshot of the NUIG_EyeGaze01 repository on Mendeley data.

**Figure 15:**
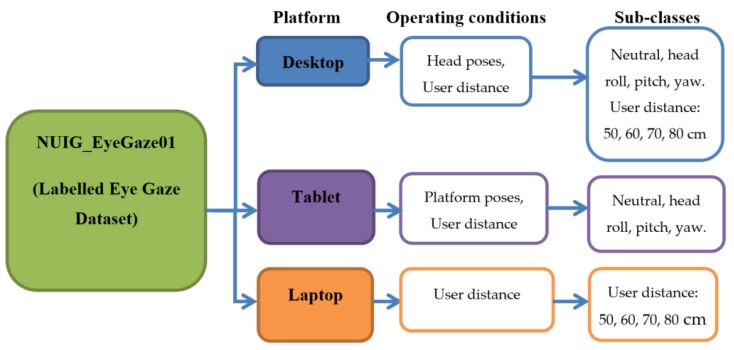
Dataset organization in the NUIG_EyeGaze01 repository on Mendeley data.

**Figure 16:**
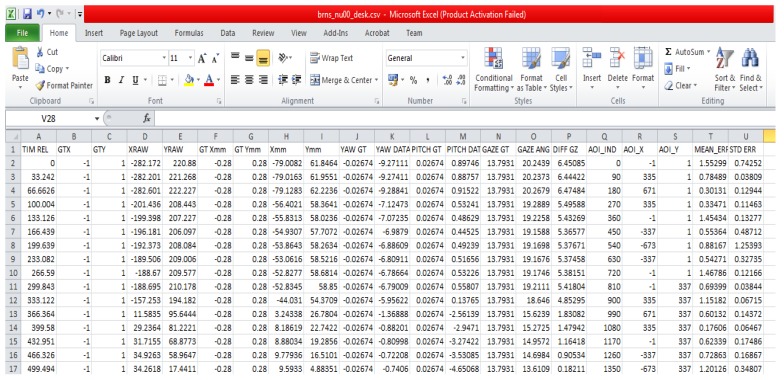
A screenshot of the data format in each comma separated values (CSV) file in the gaze dataset.

The NUIG_EyeGaze01 dataset hosted on Mendeley is shown in Figure 14. It contains gaze and ground truth data in CSV files distributed under multiple folders and subfolders which are depicted in Figure 15. Each CSV file in the dataset contains 21 columns (Figure 16) with multiple gaze data variables estimated from the raw gaze coordinates. The variables are computed from the raw gaze data using the methods described in reference [4]. Other than the raw gaze data, inputs for calculating the variables are resolution, pixel pitch of the display where gaze was tracked, and user distance from the tracker.

Within the NUIG_EyeGaze01(Labelled eye gaze dataset) data repository, the data CSV file names are labelled with the participant number, platform name and operating condition. Name of each gaze data file has the convention: USERNUMBER_CONDITION_PLATFORM.CSV (e.g., us01_80_desk.csv). The data files can be downloaded, and respective column values can be read to directly use or visualize them using Python or any CSV reading program. A detailed documentation of the data is also provided within the repository.

The NUIG_EyeGaze01 data repository is published under CC BY-NC 3.0 license. According to this license, Licensees may copy and distribute the material if they give the licensor the credits (attribution). Licensees may distribute derivative works only under a license identical to the license that governs the original work. The license also specifies that Licensees may use the data only for non-commercial purposes and there is also the condition that Licensees may copy, distribute, display, and perform only verbatim copies of the work, not derivative works of it.

There remain possibilities for extending this gaze dataset by collecting gaze data under other challenging conditions. For example, calibration could be done with a fixed head pose, and then gaze data be collected from the subject in another head pose. Then, this data could be compared with that from fixed head pose and its specific calibration. Another scenario could be collecting gaze data when head pose and eye tracker pose change together, e.g., in an automotive environment.

### 4.3. Using Data from the NUIG_EyeGaze01 Repository

**Figure 17:**
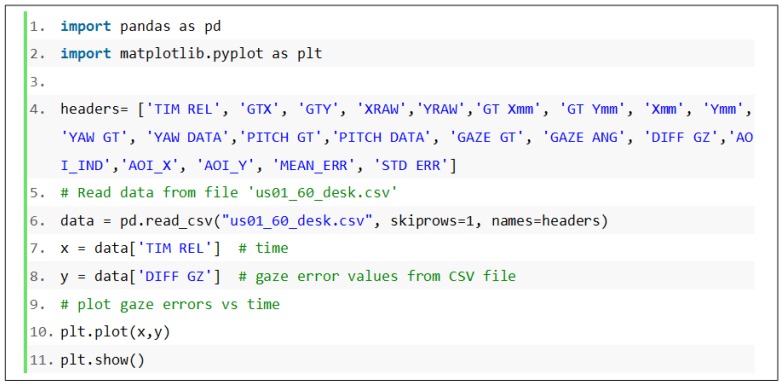
Python code snippet to read data from a gaze data file from NUIG_EyeGaze01 repository.

Users can read gaze data and other variables from any of the CSV data files present in the NUIG_EyeGaze01 repository on Mendeley Data using Python and the Pandas library (after downloading the files to their computer). Figure 17 shows such a code snippet that can be used for reading data from a gaze data CSV file and plotting the gaze error variable as a function of time.

### 4.4. Analysing Gaze Data from the NUIG_EyeGaze01 Repository

In order to study the characteristics of gaze data collected from the different eye tracker platforms (desktop, tablet) and under different operating conditions, statistical analysis is done on the datasets and their results are provided below. Table 6 and Table 7 below present the gaze error statistical values (mean, median absolute deviation, interquartile range, and 95% confidence intervals) from desktop and tablet experiments respectively. The methods for calculating gaze errors and estimating statistical metrics on gaze error values is provided in our previous paper [4]. It may be noted that the gaze data used for this analysis is available in the NUIG_EyeGaze01 data repository, and the software codes used for the gaze data analysis are provided in the GazeVisual-Lib GitHub repository.

**Figure 18:**
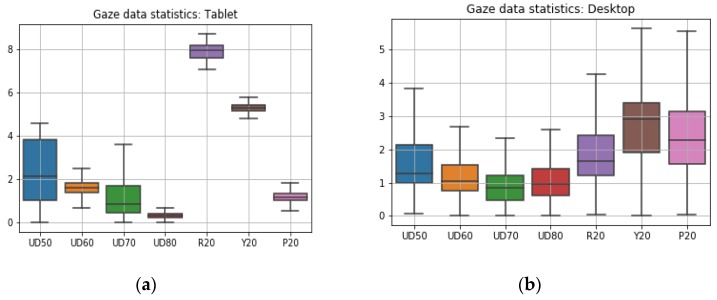
Gaze error statistics (box plots) from (**a**) desktop experiments and (**b**) tablet experiments. *Y*-axes of the plots represents gaze error in degrees. *X*-axis represents the different experiments from which data was used for plotting.

In Table 6 and Figure 18a, the terms UD 50, UD60, UD70, and UD80 correspond to gaze data from different user-distance experiments done on the desktop platform and R20, Y20, and P20 correspond to gaze data from head pose roll pitch yaw angle (20 degrees for each) experiments. All value-fields in the table have units in degrees of angular resolution. It is seen that gaze error levels are higher at low user distances and error reduces as user-tracker distance increases. Errors due to head yaw are seen to have the highest magnitude and errors due to head pitch have the highest inter-quartile range (or variability) in error magnitudes. Also, error levels due to various head poses are quite higher compared to when head pose is neutral (UD60 values in Table 6).

In Table 7 and Figure 18b, UD 50, UD60, UD70, and UD80 correspond to gaze data from different user-distance experiments done on the tablet platform and R20, Y20, and P20 represent data from the tablet pose roll pitch yaw angles (20 degrees for each) experiments. It is seen that magnitudes of gaze angular errors due to tablet pose are high, and the highest error is caused due to platform roll variations. The error characteristics from tablet data are quite different than those from the desktop platform, and error magnitudes are lower for tablet for all user distances. Also, magnitudes of errors due to different platform poses (Figure 18b) are higher than errors due to head poses (Figure 18a).

**Figure 19:**
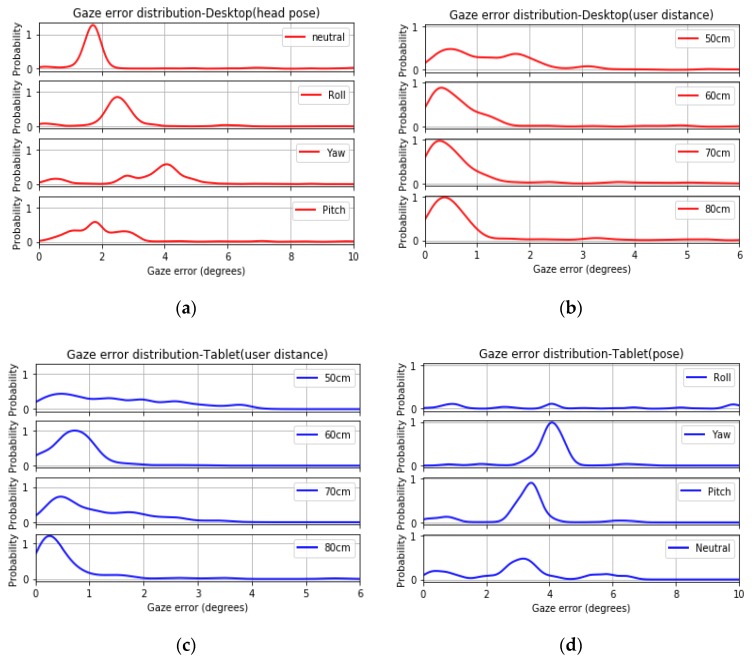
Gaze error distribution due to (**a**) user distance–desktop, (**b**) head pose–desktop, (**c**) user distance–tablet, and (**d**) platform pose–tablet.

Figure 19a,b below show gaze error distributions for the data (after outlier removal) from desktop user distance and head pose experiments. The gaze error distributions are estimated using Kernel Density Estimate [10] on gaze error values corresponding to different operating conditions, using Gaussian Kernel and a bandwidth value of 0.2. It is seen that each operating condition leaves a definite signature on the gaze error distributions. Distinction exists between patterns of gaze errors for different user distances and head poses as the error distribution shifts toward higher, average, or lower error values for different conditions. Similar observations are made for tablet data for different conditions (Figure 19c,d). The error distributions are seen to be non-Gaussian and also do not resemble any known statistical distribution.

## 5. Utility and Impact of Open Resources toward Eye Gaze Research

The GazeVisual-lib repository described in this paper provides a set of open and standardized methods for gaze data evaluation to the interdisciplinary eye gaze research community so that gaze data from a variety of eye trackers, dynamic applications [50,51,52,53], or user platforms may be evaluated and compared under a unified framework. While using the repository, users can fully understand the sequence of development of the data evaluation codes, starting from raw gaze data, making these methods adaptable to gaze data from any source. With these methods, the practical limits and capabilities of any eye tracking system may be studied and compared quantitatively and can also be upgraded by researchers to adapt to their individual research problems.

Since knowing the quality of gaze data is essential for ensuring the reliability of any gaze-based application or research, the evaluation routines of the repository can be used to constantly monitor the data quality of any eye tracker, especially during real-life operations that accompany variable setup and user conditions. Using the GUI application tool, users can perform in depth gaze data evaluation without the need for any detailed programming knowledge owing to its simple interface. This is particularly important due to the inter-disciplinary nature of gaze research where eye trackers are used widely by people from non-technological fields. The intended user group of the GazeVisual-Lib code repository is therefore quite diverse, ranging from developers of gaze estimation algorithms to users from fields like human-computer interaction, psychology and cognitive studies. Incidentally, gaze data quality is a critical aspect that affects all the stages of any gaze data–based research or application, and the open-source codes for gaze data evaluation are therefore expected to be highly useful in this respect.

The experiments described in this paper have helped to develop and introduce an accessible, diverse, and benchmark eye gaze dataset that can aid in identifying the capabilities and limits of different eye tracking systems. Such labelled gaze datasets containing signatures of different operating conditions that frequently affect gaze data quality on different user platforms do not exist yet, and keeping this in mind, the NUIG-Eyegaze01 dataset has been made publicly available. The data can be put to a wide range of uses, including modelling and comparing error patterns [54], development and testing of gaze anomaly detection algorithms, or gaze error compensation algorithms, to name a few. These are all sparsely explored areas in gaze research, which could benefit from our diverse and open data repository. Further, the datasets may also be augmented using the data augmentation routines in the GazeVisual-Lib repository. The code and data repositories are therefore complementary to each other. A major utility of presenting the data and code repositories as open resources is that they are meant to encourage research toward practical and realistic performance evaluation of eye trackers, standardization of gaze research results, and building of more open-source tools for these purposes.

## 6. Conclusions

The open-source gaze data evaluation methods of GazeVisual-Lib could be useful for researchers, engineers, and developers working with gaze estimation systems for the thorough assessment of their gaze data quality. The methods could be especially beneficial for eye trackers that operate under variable operating conditions where gaze data quality frequently becomes unreliable. Also, the GUI application GazeVisual may be used to perform prompt and in-depth gaze data evaluation without the need for any detailed programming knowledge. This could be particularly useful for the inter-disciplinary gaze research community where eye trackers are used widely in non-technological fields. The potential user group of GazeVisual-Lib is therefore quite diverse, ranging from gaze tracking system developers, researchers using eye trackers in virtual/augmented reality, human–computer interactions, cognitive sciences, and generic users having any consumer-grade eye tracker or gaze-based application.

The new eye gaze database NUIG_EyeGaze01 presented in this paper could be beneficial to designers of gaze-based systems for benchmark comparison of their system performances under challenging operating conditions such as variations of head pose, user distance, and tracker orientations. As can be observed from the gaze data analysis results presented in Section 4.4, possible future directions of research using these gaze datasets (in conjunction with the coding resources of GazeVisual-Lib) include comparison of gaze error patterns from multiple eye trackers, modelling of gaze error patterns induced by different operating conditions, studying gaze error distributions, or the development of gaze error pattern detection algorithms. These would depend on how gaze researchers, statisticians, and researchers working with machine learning models would prefer to use these datasets. The open resources presented in the paper are envisioned to foster collaborative development and adoption of even better resources toward standardized gaze data evaluation, which ultimately can strengthen the usability and reliability of gaze estimation systems in their wide range of applications.

## Figures and Tables

**Table 1:** Eye gaze datasets for building gaze estimation algorithms.

Dataset Name	Type	Purpose of the Dataset	Description
CAVE [22]	images	Dataset built to train a detector to sense eye contact in an image. Can be used for gaze estimation or tracking	56 subjects (32 male, 24 female). 5880 images, acquired for combinations of five calibrated head poses. Dataset includes users wearing glasses.
Weidenbacher et al. [23]	images	Evaluations of computational methods for head pose and eye gaze estimation. Benchmark dataset for Point of Gaze (PoG) detection algorithms	20 subjects, 2220 color images. Various head poses, nine different gaze conditions for each head pose. Participants with glasses included.
McMurrough et al. [24]	videos	Training and testing data for appearance-based gaze estimation methods.	20 subjects. Videos recorded as subjects followed a set of predefined points on a screen. Participants didn’t wear spectacles, free head motion.
UT Multiview [25]	images	Building and testing 3D gaze estimation algorithms	50 (15 female and 35 male) subjects. 160 gaze directions per person were acquired using eight cameras. 64,000 eye images, 8000 3D eye shape models, 1,152,000 gaze directions.
MPII Gaze [26]	images	For appearance-based gaze estimation in the wild.	15 participants. 213,659 images. some samples manually annotated with 6 facial landmarks and pupil centers. Free head motion, uncontrolled illumination.
OMEG: Oulu Multi-Pose Eye Gaze Dataset [27]	images	Evaluating and comparing gaze tracking algorithms.	50 subjects. Over 40,000 images captured under fixed and free head poses. Five landmark labels and gaze angles are provided as ground truth.
MSP Gaze corpus [28]	videos	For appearance-based, user dependent, or independent gaze estimators.	46 subjects. Videos with/without head movement, different user distance, free head motion.
EYEDIAP [29]	videos	For training and evaluation of gaze estimation algorithms from RGB and RGB-D data.	16 subjects (12 male, four female). 94 session recordings, each with different characteristics of ambient conditions and types of targets.
3D mask attack dataset [30]	videos	Testing biometric face spoofing attacks.	17 subjects. 76,500 frames recorded using Kinect for. Eye-positions manually labelled in each video.
HPEG [31]	videos	For testing head pose and eye gaze estimation algorithm.	10 subjects (two female, eight male). Free movement of subjects, 20 color video sequences.
I2Head [32]	videos	Reference dataset for low-cost gaze estimation.	12 subjects. Dataset contains head pose, gaze and user face models. Webcam is and head pose sensors used.

**Table 2:** Eye gaze datasets for saliency/cognitive studies.

Dataset Name	Description
Hadizadeh et al. [33]	Video database for computational models of visual attention. Twelve video sequences with eye-tracking data. Gaze fixations recorded using a head-mounted eye-tracker 15 participants (two women and 13 men).
DOVES [34]	Benchmark for testing gaze modelling algorithms. Contains fixation coordinates and eye movement trajectories of 29 observers as they viewed 101 natural calibrated images and 30,000 fixation points.
Fixations in Faces (FiFA) [35]	Recorded to demonstrate that faces attract significant visual attention while viewing images through free-viewing, search, and memory tasks.
KTH Eye-tracking Dataset [36]	Comprises of complex photographic images and was used to validate a saliency model predicting interesting image regions. The study concluded that early eye fixations are observed in symmetrical image areas.
McGill ImgSal [37]	Aims to validate a frequency domain-based saliency detector incorporating scale-space analysis.
MIT CSAIL Saliency [38]	Publicly available, large-scale eye movement database to aid natural image-related visual attention studies. Used to validate a supervised saliency model combining top-down/ bottom-up cues.
MIT Low-Resolution Saliency [39]	Compiled to study how image resolution affects consistency in eye fixations across observers. The study noted that eye fixations are biased towards the image center for all resolutions.
NUS Eye Fixation (NUSEF) [40]	Contains a repository of eye fixations to study viewing patterns on semantically rich and diverse images, including faces, portraits, indoor/outdoor scenes, and affective content.
Toronto Dataset [41]	Contains eye movement recordings while viewing natural scenes to validate a visual saliency model based on the principle of maximizing scene information.
Visual Attention for Image Quality-VAIQ [42]	Provides eye-tracking data for reference images from three image quality databases to validate the hypothesis that salient image regions should contribute more to objective image quality metrics.
Actions in the Eye Dataset [43]	Two subject groups—an active group of 12 subjects performed action recognition, while a second group of four subjects free-viewed the videos. Fixation patterns of free and active.
SALICON [44]	Saliency in Context eye tracking dataset, 1000 images with eye-tracking data in 80 image classes.
DR(EYE)VE [45]	Seventy-four video sequences of 5 min each, captured and annotated more than 500,000 frames. The labeling contains drivers’ gaze fixations and their temporal integration.
OSIE [46]	Seven hundred images, 5551 segmented objects, eye tracking data.
CITIUS [47]	A database of 72 videos with eye-tracking data to evaluate dynamic saliency visual models.
Eye Movements in Programming (EMIP) [48]	The EMP dataset contains eye movement data during program comprehension data on eye movement parameters such as horizontal and vertical pupil positions, pupil center, diameter, corneal reflex positions, gaze vector, and point of regard, along with programming experience of participants in various languages.

**Table 3:** Features of GazeVisual-Lib repository.

Repository Parameters	Description
File types in repository	py and .IPYNB (Python scripts and Jupyter notebooks)
Link to repository	https://github.com/anuradhakar49/GazeVisual-Lib
Legal Code License	GNU General Public License v3.0
Software languages used	Python
Operating environments & dependencies	Operating environment: Python 2.7; Dependencies: Python libraries Tkinter, Pygame, Statsmodels, Seaborn, CSV, Pandas, Sklearn. Scipy, Numpy, Matplotlib, PIL
Latest version date	July 2019

**Table 4:** Features of the gaze data collection process for building the NUIG_EyeGaze01 dataset.

Parameter	Description
Data type	Fixations
Data file type	CSV
Eye tracker	Tobii EyeX 4C with specified accuracy of 0.5 degrees.
Data collection platforms	Desktop, tablet, laptop
Screen sizes and resolutions	Desktop: 22 inch diagonal, 1680 × 1050 pixels
	Laptop: 14 inch diagonal, 1366 × 768 pixels
	Tablet: 10.1 inch diagonal, 1920 × 800 pixels
Visual stimuli details	Moving dot stopping for 3 s at 15 locations on display screen. Stimulus dot size 10 pixels, color black.
Number of Participants	Twenty for all experiments, 15 male, five female, no glasses.
Ambient illumination	Constant, 60 Lux, Indoor setup.
Chin rest	Used for all sessions, Head poses measured using webcam with real time head pose model.
Desktop data details	Gaze data for four user distances: 50, 60, 70, 80 cm
	Gaze data for following head poses: Neutral, roll plus (10, 20, 30 degree),
	roll minus (10, 20, 30 degree)
	Neutral, pitch plus (10, 20 degree),
	pitch minus (10, 20 degree)
	Neutral, yaw plus (10, 20, 30 degree),
	yaw minus (10, 20, 30 degree)
Tablet data details	Gaze data for four user distances: 50,60, 70, 80 cm
	Gaze data for following platform poses:
	Neutral, roll plus 20 degree, minus 20 degree
	Neutral, pitch plus 10 degree, plus 20 degree
	Neutral, yaw plus 20 degree, minus 20 degree
Laptop data details	Gaze data for four user distances: 50, 60, 70, 80 cm

**Table 5:** Columns in a gaze data file from the NUIG_EyeGaze01dataset and their meaning.

Parameter	Description
“TIM REL”	Relative time stamp for each gaze data point in the file (measured during data collection)
“GTX”, “GTY”	Ground truth x, y positions of stimuli in pixels
“XRAW”, “YRAW”	Raw gaze data x, y coordinates in pixels
“GT Xmm”, “GT Ymm”	Ground truth x, y positions in mm, converted using the pixel pitch value
“Xmm”, “Ymm”	Gaze x, y positions in mm, converted using the pixel pitch value
“YAW GT”, “YAW DATA”	Ground truth and estimated yaw angles from input gaze data
“PITCH GT”, “PITCH DATA”	Ground truth and estimated pitch angles from input gaze data
“GAZE GT”, ”GAZE ANG”	Ground truth and estimated gaze primary angles from input gaze data
“DIFF GZ”	Difference between ground truth and estimated gaze primary angles, i.e., Gaze angular accuracy, Index of each stimulus point
“AOI_X”, ”AOI_Y”	Index of each stimulus position X, Y coordinates of each stimulus position
“MEAN_ERR”, “STD ERR”	Mean and standard deviation of gaze estimation error at each stimulus position

**Table 6:** Gaze error statistics from desktop experiments (table values in degrees).

	UD50	UD60	UD70	UD80	Roll 20	Yaw 20	Pitch 20
Mean	3.37	2.04	1.21	1.02	3.7	8.51	3.15
MAD	3.49	1.77	0.82	0.66	3.63	10.0	1.90
IQR	1.13	0.77	0.76	0.79	1.21	1.49	1.59
95% interval	3.15–3.59	1.90–2.18	1.15–1.26	1.16–1.24	3.30–4.09	7.60–9.43	2.83–3.47

**Table 7:** Gaze error statistics from tablet experiments (table values in degrees).

	UD50	UD60	UD70	UD80	Roll 20	Yaw 20	Pitch 20
Mean	2.68	2.46	0.59	1.55	7.74	4.25	2.45
MAD	0.38	0.42	0.29	0.24	0.77	0.60	0.46
IQR	0.39	0.54	0.33	0.22	0.75	0.53	0.23
95% interval	2.65–2.71	2.43–2.48	0.57–0.61	1.53–1.57	7.69–7.80	4.22–4.29	2.41–2.49

**Figure A1:**
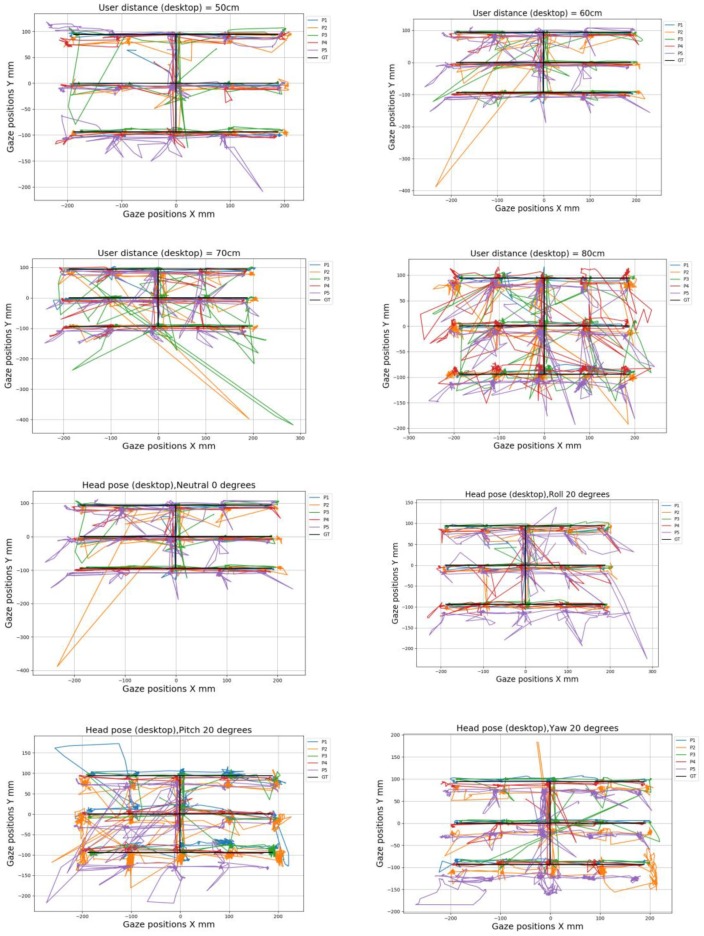
Gaze data from desktop-based user distance and head pose experiments.

**Figure A2:**
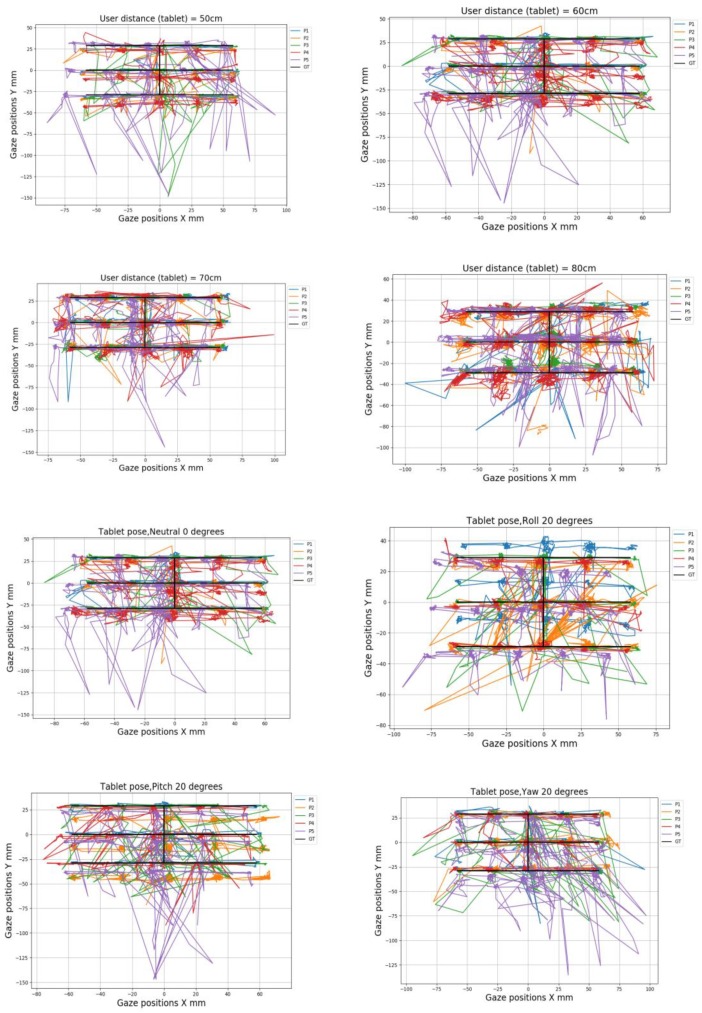
Gaze data from tablet-based user distance and tablet pose experiments.

## Supplementary Materials

The following are available online, Video S1: https://www.youtube.com/watch?v=mPGlw711BCA. Video S2: https://www.youtube.com/watch?v=sir_qZmvGME.

## Appendix A

**Table A1:** Python libraries to be installed for using GazeVisual-Lib and their commands.

No.	Python Library Name	Anaconda/Pip Command
1.	matplotlib	conda install -c conda-forge matplotlib pip install matplotlib
2.	PIL	conda install -c anaconda pil, pip install Pillow==2.2.2
3.	ttk	conda install -c conda-forge pyttk, pip install pyttk
4.	pandas	conda install -c anaconda pandas, pip install pandas
5.	statsmodels	conda install -c anaconda statsmodels pip install statsmodels
6.	seaborn	conda install -c anaconda seaborn pip install git+https://github.com/mwaskom/seaborn.git#egg=seaborn
7	pygame	conda install -c cogsci pygame pip install pygame
8.	scipy	conda install -c anaconda scipy pip install scipy
9.	sklearn	conda install -c anaconda scikit-learn pip install -U scikit-learn
10.	itertools	conda install -c conda-forge more-itertools pip install more-itertools
11.	Git	conda install -c anaconda git, pip install python-git
12.	Jupyter notebook (comes by default with Anaconda distribution)	pip install jupyter

## Appendix B

Gaze data (on-screen x, y coordinates) of five participants (P1–P5) belonging to different desktop and tablet-based experiments are presented below along with ground truth data (GT, in black). By ground truth we mean the screen location (x, y coordinates) of the stimuli dots as they appear on the screen while the user looks at them. The gaze data collection procedure has been discussed in detail above in Section 4.1. During experiments, a visual stimulus in the form of a black moving dot sequentially moves over a grid of (5 × 3) locations over the display screen (of desktop or tablet). The screen locations traced by the stimulus dot are shown as the black lines in plots B.1 and B.2 which is our ground truth data. The gaze data comprises of a participant’s gaze coordinates on the display as they follow the stimuli dots. It may be noted that our Tobii tracker operates at 30 fps and therefore cannot register rapid eye movements like drifts, saccades. and micro-saccades.
